# Realistic Worst Case for a Severe Space Weather Event Driven by a Fast Solar Wind Stream

**DOI:** 10.1029/2018SW001948

**Published:** 2018-09-03

**Authors:** Richard B. Horne, Mark W. Phillips, Sarah A. Glauert, Nigel P. Meredith, Alex D. P. Hands, Keith A. Ryden, Wen Li

**Affiliations:** ^1^ British Antarctic Survey Cambridge UK; ^2^ Surrey Space Centre University of Surrey Guildford UK; ^3^ Center for Space Physics Boston University Boston MA USA

**Keywords:** radiation belts, worst case, fast solar wind, ESD, geostationary orbit

## Abstract

Satellite charging is one of the most important risks for satellites on orbit. Satellite charging can lead to an electrostatic discharge resulting in component damage, phantom commands, and loss of service and in exceptional cases total satellite loss. Here we construct a realistic worst case for a fast solar wind stream event lasting 5 days or more and use a physical model to calculate the maximum electron flux greater than 2 MeV for geostationary orbit. We find that the flux tends toward a value of 10^6^ cm^−2^·s^−1^·sr^−1^ after 5 days and remains high for another 5 days. The resulting flux is comparable to a 1 in 150‐year event found from an independent statistical analysis of electron data. Approximately 2.5 mm of Al shielding would be required to reduce the internal charging current to below the National Aeronautics and Space Administration‐recommended guidelines, much more than is currently used. Thus, we would expect many satellites to report electrostatic discharge anomalies during such an event with a strong likelihood of service outage and total satellite loss. We conclude that satellites at geostationary orbit are more likely to be at risk from fast solar wind stream event than a Carrington‐type storm.

## Key Points

- We calculate the electron flux at geostationary orbit for a realistic worst‐case event
- Approximately 2.4 mm of Al is required to shield electronic components
- Satellites at geostationary orbit are more at risk from a fast solar wind stream event than a Carrington‐type event

## 1 Introduction

Our reliance on satellites for applications such as mobile phones, broadcasting, navigation, and timing signals is growing substantially. New methods of launching satellites using electric orbit raising (Horne & Pitchford, 2015; Wade et al., 2015) and registered plans to launch constellations of hundreds of satellite for Internet services (e.g., http://oneweb.net/solution) show that the space sector is innovating and developing rapidly. As a result there has been a growing concern about the impact of a severe space weather event and the disruption it could cause. A recent study found that 10% of the entire satellite fleet suffered anomalies (malfunctions) during the so‐called Halloween storm of 2003 (Cannon et al., 2013) resulting in loss of service and in one case total satellite loss. Assuming that the same percentage of satellites would be affected, approximately 150 spacecraft would be disrupted if a similar storm took place today. The 2003 Halloween storm was by no means as big as the 1859 Carrington storm, and so for an extreme event the number of satellites affected could be much higher, but remains very uncertain.

Internal satellite charging is one of the most important hazards for satellites on orbit (Fennell et al., 2012; Gubby & Evans, 2002; Hastings & Garrett, 1996; Wrenn & Smith, 1996). During a space weather event the flux of high‐energy relativistic (>2 MeV) electrons in the outer radiation belt can increase by up to 5 orders of magnitude (Baker et al., 2007). These relativistic electrons can penetrate the outer surface of the spacecraft and cause charge accumulation on dielectrics such as cables and circuit boards as well as on any ungrounded conductors internal to the spacecraft. If the flux remains high for a substantial period of time, the accumulated charge can break down the dielectric and cause an electrostatic discharge (ESD) resulting in a satellite anomaly. Several studies have shown that satellite anomalies are related to an enhanced electron flux at energies typically greater than 2 MeV (Baker, 2001; Iucci et al., 2005; Wrenn, 1995). Furthermore, it is not just the instantaneous flux that is important but the time history of the flux that causes charge accumulation in dielectrics with a long time constant (Bodeau, 2010). Satellite anomalies due to ESD can disrupt operations and have resulted in loss of service, and in some cases total satellite loss, resulting in insurance claims (Horne, Glauert, et al., 2013; Wade et al., 2015).

There is more than one type of event on the Sun that can lead to severe space weather at Earth. Probably, the most well known is a coronal mass ejection (CME) leading to a major geomagnetic storm. CMEs are believed to be responsible for the 1859 Carrington storm (Tsurutani et al., 2003) and the March 1989 storm (Allen et al., 1989), which caused widespread disruption (Hapgood, 2012). Two or more CMEs following in quick succession can cause multiple geomagnetic storms and may also result in severe space weather. In fact the Carrington storm and the so‐called Halloween storm of October and November 2003 were the result of two CMEs (Webb & Allen, 2004).

Perhaps less well known but just as important are fast solar wind stream events. Fast solar wind streams emanate from coronal holes on the Sun (Krieger et al., 1973). They do not compress the magnetosphere significantly nor do they cause major geomagnetic storms, but they can result in severe space weather if the interplanetary magnetic field is predominantly southward (e.g., Miyoshi et al., 2007; Tsurutani et al., 2006). The combination of high solar wind speed and southward fluctuations in the interplanetary magnetic field is associated with a high flux of relativistic electrons in Earth's radiation belts for periods of several days or more (e.g., Lam et al., 2009; Meredith et al., 2011; Tsurutani et al., 2006). These events pose a risk of satellite damage due to ESD as a result of internal satellite charging. For example, Intelsat K, Anik E1, and Anik E2 experienced anomalies on 20 January 1994 associated with a sustained high flux of relativistic electrons at geostationary orbit during a fast solar wind stream event (Baker, 2001; Lam et al., 2012). Fast solar wind streams also have the potential to disrupt ground‐based systems such as power grids since they cause multiple substorms. Substorms drive strong variable currents in the auroral electrojet and hence strong variations in the horizontal component of the magnetic field *d* *B*_*H*_/*d* *t*, which drives geomagnetically induced currents in ground conductors (Thomson et al., 2011).

In order to assess the impact of a severe space weather event on the existing satellite fleet, and to develop new mitigation measures, satellite designers, operators, and space insurers need to know the reasonable worst case. Statistical methods have been used to calculate the extreme electron flux for satellites in geosynchronous orbit (Koons, 2001; Meredith et al., 2017; O 'Brien et al., 2007), low Earth orbit (Meredith, Horne, Isles, & Green, 2016) and highly elliptical orbit (Meredith et al., 2017; O 'Brien et al., 2007) and to calculate internal charging currents along the Galileo satellite orbits (Meredith, Horne, Isles, Ryden, et al., 2016). Most of these studies have been limited to just one or two solar cycles due to the availability of data. They also work on the assumption that the system is stationary in time. Based on historical auroral records the return period of a Carrington‐type event is about 150 years (Lloyds, 2013), and statistical studies have been able to provide a valuable calculation of the electron flux for this return period. However, as the data used in these studies is somewhat limited compared to a return period of 150 years, and does not include an extreme event of the size of the Carrington event, one is still left wondering if the flux could be higher.

There have been a number of other attempts to calculate a limiting electron flux based on the idea of a stably trapped limit obtained by balancing energy lost via wave propagation against the growth of plasma waves (Kennel & Petschek, 1966; Mauk & Fox, 2010; Schulz & Davidson, 1988; Summers et al., 2009). However, these studies did not take into account the acceleration of electrons by resonant wave‐particle interactions, which are now known to be very important for energies of several hundred kiloelectron volts or more.

Here we present an alternative approach. Over the last few years our physical understanding of the processes controlling the variability of the radiation belts has developed considerably. For example, we now know that cyclotron resonant wave‐particle interactions, substorms, and ultralow frequency (ULF) waves play a major role in the acceleration, transport, and loss of relativistic electrons. The purpose of this paper is to use this new physical understanding to calculate the maximum electron flux at geostationary orbit for a severe space weather event driven by a fast solar wind stream. We have selected a fast solar wind steam event rather than a CME event as it is probably one of the least well‐recognized types of severe space weather and, since fast solar wind streams do not compress the magnetosphere inside geostationary orbit, it is more likely to cause internal satellite charging around the entire geostationary orbit.

The paper is organized as follows. In section 2 we present an example of fast solar wind stream events to illustrate their effects on the radiation belts and provide the general concept behind our work. In section 3 we describe a model to calculate the maximum electron flux for geostationary orbit based on electron acceleration by wave‐particle interactions. In section 4 we define our reasonable worst‐case scenario, and in section 5 we calculate the maximum electron flux. In section 6 we test the sensitivity of the results to changes in the space environment, and in section 7 we calculate the amount of shielding required to reduce the risk of an ESD according to the National Aeronautics and Space Administration (NASA) design guidelines. Section 8 includes a discussion of other factors, and the summary and conclusions are given in section 9.

## 2 Fast Solar Wind Stream Events

**Figure 1:**
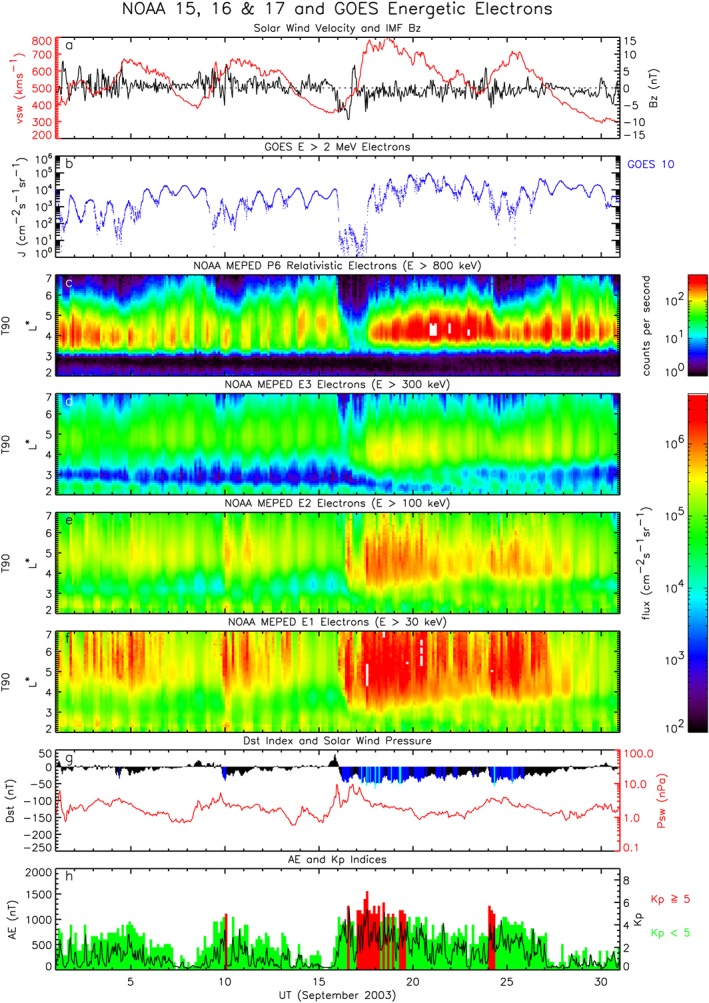
Example of four fast solar wind stream events observed during September 2003. Shown are (a) the solar wind speed (in red) and IMF B_z_ (in black) measured by the ACE spacecraft in the solar wind at the L1 position, (b) the >2‐MeV electron flux measured by GOES at geosynchronous orbit, (c) counts measured by the T90 P6 proton detector on the low‐altitude Polar Operational Environmental Satellites, which is a measure of electrons >800 keV, (d) the electron flux >300 keV measured by the T90 E3 detector, (e) the electron flux >100 keV measured by T90 E2, (f) the electron flux >30 keV measured by T90 E1, (g) solar wind dynamic pressure (in red) and D_st_ index (color coded: black, >−30 nT; blue, −30 to −50 nT; and light blue −50 to −100 nT), and (h) the A E index (black line) and K_p_ index (color coded). GOES = Geostationary Operational Environmental Satellite; IMF = interplanetary magnetic field; NOAA = National Oceanic and Atmospheric Administration.

Figure 1 shows an example of four fast solar wind stream events that occurred in September 2003. We should emphasize that these are not extreme events, but they are shown here to illustrate the characteristics. Figure 1a shows the solar wind speed measured by the Advanced Composition Explorer spacecraft in the solar wind at the L1 position. The solar wind velocity increased above 500 km/s on 4, 9, 17, and 23 September 2003. Typically each event lasted for a few days, with the longest event starting on 17 September 2003 and lasting 6 days. During each event there was in general an increase in the integral electron flux greater than 2 MeV, (*J*(*E* > 2)) measured by Geostationary Operational Environmental Satellite (GOES) at geostationary orbit (Figure 1b). The largest electron flux exceeded 10^5^ cm^−2^·s^−1^·sr^−1^ around 20 and 21 September. The daily variation in the flux is attributed to the drift of electrons around the Earth in a nondipolar magnetic field, which does not coincide with geosynchronous orbit. The flux is highest on the dayside near noon. The increase in flux was also detected by the Polar‐orbiting Operational that are in a high‐inclination low Earth orbit at approximately 800‐km altitude. For example, Figure 1c shows the counts in the proton detector that, in the absence of any high proton flux, is sensitive to electrons greater than approximately 800 keV (Rodger et al., 2010; Sandanger et al., 2007). The data show that the electron flux is highest between *L* ^∗^=4 and 5, corresponding to medium Earth orbit, but is also elevated at geostationary orbit near *L* ^∗^=6. Note that the flux is also significantly enhanced at the lower energies >300, >100, and >30 keV (Figures 1d–1f) indicating multiple injections of lower‐energy electrons associated with substorms.

During the fast solar wind stream events the *D*_*st*_ index (Figure 1g) did not drop much below −50 nT indicating that there were no significant magnetic storms. However, each event is accompanied by a significant increase in the *A* *E* index (Figure 1h), which is a measure of the intensity of the auroral current systems and an indicator of substorms. The *K*_*p*_ index (Figure 1h) is also generally enhanced during the events, indicating that these are geomagnetically disturbed periods.

A full understanding as to the cause of these electron flux increases has been a matter of scientific debate for several years. However, a consensus has now emerged that electron acceleration by wave‐particle interactions plays a major role in the formation of the Earth's radiation belts (Horne, Thorne, Glauert, et al., 2005; Horne, Thorne, Shprits, et al., 2005; Horne, 2007; Li et al., 2016; Reeves et al., 2013; Thorne et al., 2013; Tu et al., 2014). In general, electrons at energies of a few kiloelectron volts up to a few hundred kiloelectron volts are injected toward the Earth on the nightside during substorms. At these energies the electrons drift around the Earth through dawn to the dayside under the driving force of the convection electric field (e.g., Jordanova et al., 2016). Some of the injection events can be very narrow in local time. These injected electrons form a source population that excites plasma waves and in particular chorus plasma waves (e.g., Li et al., 2009). Chorus waves react back on the electron distribution and precipitate some of the lower‐energy electrons into the atmosphere causing loss (Lam et al., 2010). However, they also accelerate a fraction of the population to very high energies, typically a few megaelectron volts, which remain trapped in the geomagnetic field (Horne & Thorne, 1998; Horne, Thorne, Glauert, et al., 2005; Horne, Thorne, Shprits, et al., 2005; Thorne et al., 2013; Summers et al., 1998). These higher‐energy electrons are transported (diffused) across the geomagnetic field toward and or away from the Earth by ULF waves to form the entire radiation belts (Elkington et al., 1999; Mann et al., 2004). There are many other types of plasma waves and ULF waves that take part in the interaction, and other processes that contribute, but this is the essence of our current understanding.

## 3 Method

In order to calculate the increase in the electron flux we used a two‐dimensional version of the British Antarctic Survey radiation belt model (Glauert et al., 2014). This model treats the radiation belts as a diffusion problem and solves the Fokker‐Planck equation to obtain the time evolution of the electron distribution function and hence the electron flux. Here the distribution is a function of pitch angle and energy. More details are given in Appendix A. The effects of resonant wave‐particle interactions are included as diffusion coefficients, and they in turn require information on the plasma wave properties and the plasma density. It is these wave‐particle interactions, primarily with lower‐band chorus waves, that cause the increase in the electron flux in our model. Nonlinear particle trapping effects by plasma waves are omitted in this approach. The British Antarctic Survey radiation belt model has been used to reconstruct the last 30 years of the radiation belts and has been shown to have a skill score against persistence of between 0.6 and 0.8, where 1 would be a perfect prediction and 0 effectively says that tomorrow is the same as today (Glauert et al., manuscript submitted, 2018). Thus, a skill score greater than 0.5 suggests that the model performs very well.

Electron acceleration by lower‐band chorus waves is the primary interaction in these calculations since they accelerate electrons up to several megaelectron volts. Other types of waves, such as magnetosonic waves (Horne et al., 2007), can also contribute to electron acceleration but have yet to be shown to be as effective as chorus. Upper‐band chorus waves and electrostatic waves resonate with lower‐energy electrons (Thorne et al., 2010), while electromagnetic ion cyclotron waves tend to resonate with much higher energy electrons (Albert, 2003; Summers & Thorne, 2003) and then only contribute to loss, not acceleration (Horne & Thorne, 1998).

Electrons are lost from the radiation belts into the atmosphere when the waves diffuse electrons into the loss cone. At geostationary orbit the loss cone angle is typically 2° wide. In the model electrons were removed from the loss cone on a timescale of a quarter of the electron bounce time, which is typically *τ*_*b*_/4 = 0.1 s at *L* ^∗^=6 at 1 MeV but varies with energy.

Radial diffusion driven by ULF waves is not included in these calculations. We have effectively assumed that the gradient in electron phase space density is flat with radial distance, so that transport across the magnetic field by radial diffusion is very efficient and removes any gradients. This is commonly observed during periods of frequent particle injections near geostationary orbit (Selesnick & Blake, 1997). The implications of this are discussed below.

## 4 Defining a Reasonable Worst Case

### 4.1 Event Duration

Figure 1a shows that when the solar wind velocity exceeded 700 km/s on 17 September it did so for 3.5 days. During this time the *A* *E* index exceeded 750 nT almost continuously. More intense but shorter duration events (>2 days) known as high‐intensity long‐duration continuous auroral activity events have also been reported (Tsurutani & Gonzalez, 1987). Other statistical analyses show that fast solar wind stream events, when they encounter the Earth, typically last 5 days or so (Denton & Borovsky, 2012; Meredith et al., 2011). Therefore, we have chosen as our reasonable worst case an event lasting 5 days with velocity exceeding 700 km/s and *A* *E* > 750 nT. This is followed by a period of another 5 days where the velocity and activity level are much reduced to test how long it takes for the electron flux to decay. More details are given below in section 5.

### 4.2 Plasma Wave Model

Observations suggest that chorus wave amplitudes are enhanced during a substorm injection and decay subsequently (Meredith et al., 1999, 2000). Since fast solar wind stream events are characterized by multiple substorms we must try to take this into account. However, it is very difficult to measure the period of time chorus is enhanced above a given level and how long it takes for the wave amplitudes to decay. This is because the waves vary so much in space and time and since satellites only spend a limited time in the source region, typically from seconds to tens of minutes. For example, while energetic electrons drift around the Earth on a timescale of 10 min or more and can be observed at different magnetic local time (MLT) if we compute the right drift path, chorus waves tend to propagate along the magnetic field and vary considerably in space and time. Thus, to overcome this problem the waves are usually treated statistically and a proxy is used to help define the wave amplitudes.

Over a number of years we have constructed a database of chorus plasma waves using data from seven different satellites. The database takes into account the variability of the waves by binning the data in MLT, *L* shell, magnetic latitude *λ*_*m*_, geomagnetic activity such as the *A* *E* and *K*_*p*_ indices (e.g., Meredith et al., 2012, 2014) and other wave properties (Horne, Kersten, et al., 2013). For this study the database was augmented with 34 months of wave data from the Electric and Magnetic Field Instrument Suite and Integrated Science waveform receiver (Kletzing et al., 2013), on the Van Allen Probes satellites. Since fast solar wind streams are characterized by continuously high levels of the *A* *E* the *A* *E* index provides a useful proxy linking fast solar wind streams to chorus wave power.

**Figure 2:**
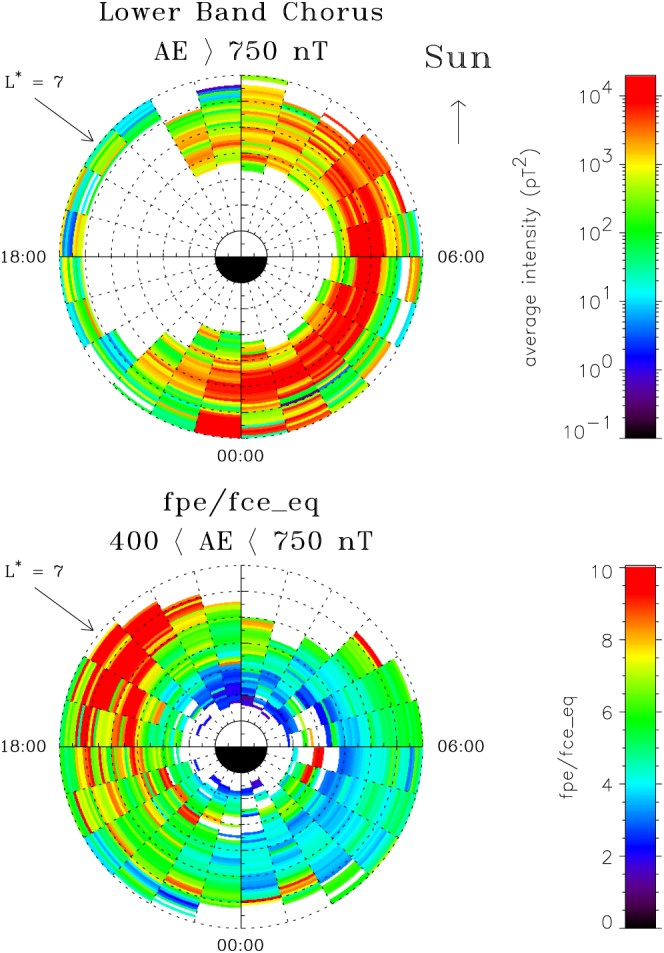
Global distribution of wave and plasma properties. (top) Average lower‐band chorus (f_LHR_<f/f_ce_<0.5) wave intensities for |λ_m_|<30° for active geomagnetic conditions corresponding to A E > 750 nT and (bottom) the global distribution of f_pe_/f_ce_ at the equator for 400 < A E < 750 nT.

Figure 2 (top) shows how the spatial distribution of lower‐band chorus waves (*f*_*LHR*_<*f* < 0.5*f*_*ce*_ where *f*_*LHR*_ is the lower hybrid resonance frequency and *f*_*ce*_ is the *equatorial* electron gyro frequency) for the highest level of *A* *E* (>750 nT) is enhanced from midnight through dawn to the dayside magnetosphere. This corresponds to the drift of lower‐energy electrons that excite chorus waves under the driving force of the convection electric field. The waves are most intense just inside geostationary orbit, which is at approximately *L* ^∗^=6. Thus, electron acceleration should be strongest for about 12 hr of MLT around the geostationary arc.

Figure 2 (bottom) shows that during high levels of geomagnetic activity the ratio of the electron plasma frequency to gyro frequency *f*_*pe*_/*f*_*ce*_ is lowest near dawn. This enables electron acceleration to higher energies by the waves (Horne et al., 2003). Data from the plasma wave instrument (Anderson et al., 1992) on the Combined Release and Radiation Effects Satellite (CRRES) were used to measure *f*_*pe*_ since CRRES had the advantage of being able to observe *f*_*pe*_ directly from the wave experiment and thus provide an accurate measurement. The data were also limited to 400 < *A* *E* < 750 nT as the number of samples for higher levels of activity were too sparse. The median and the average value between 5.5 < *L* ^∗^<6.5 and 00:00–09:00 MLT were *f*_*pe*_/*f*_*ce*_=5.16 and 5.37, respectively (*f*_*ce*_=4.04 kHz at the equator), indicating a near Gaussian distribution. Thus, we used *f*_*pe*_/*f*_*ce*_=5.37 to calculate the diffusion rates.

To define the wave spectrum for a reasonable worst case we used the average shape of the observed power spectrum near *L* ^∗^=6 and scaled it by the wave intensity. Details of the wave model are given in Appendix B. To find the chorus wave intensity we calculated the cumulative probability distribution for lower‐band chorus waves observed between 00:00 and 12:00 MLT, |*λ*_*m*_|<30° and 5.5 < *L* ^∗^<6.5 for the two highest levels of geomagnetic activity, namely, 400 < *A* *E* < 750 nT and *A* *E* > 750 nT. The median amplitudes corresponding to the median intensity were 27.8 and 31 pT, respectively, but the root‐mean‐square amplitudes were much higher (39.1 and 55 pT). As we are already selecting a period of very high *A* *E* we used the median value (31 pT) rather than the root mean square since the median is not skewed so much by extremely large values, particularly for *A* *E* > 750 nT. Finally, after calculating the diffusion coefficients the coefficients were scaled by the wave intensity and divided by a factor of 2 since the electrons only encounter the waves for approximately 50% of the drift orbit (Figure 2, top).

### 4.3 Substorms

To model a fast solar wind stream event we must define the initial electron flux at low energies. In practice the low‐energy flux varies due to the injection of electrons during substorms and a contribution due to radial diffusion. Statistical analysis shows that the most probable time between substorm onsets is 2.75 hr (Borovsky et al., 1993). The data also show that the integral flux over the energy range 140–300 keV can vary by a factor of 100 or more and then decay on a timescale of a few hours.

**Figure 3:**
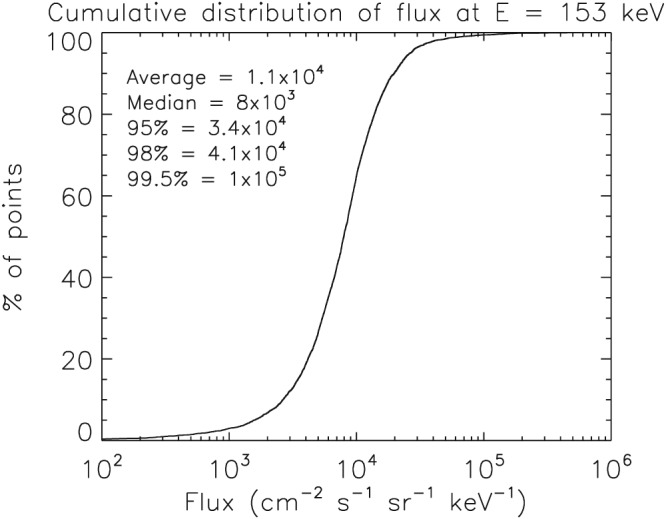
The cumulative distribution of the electron flux at 153 keV measured by the Combined Release and Radiation Effects Satellite for 5.5 < L ^∗^<6.5 and λ_m_<15° for the entire mission.

To capture the effects of electron injections we calculated the cumulative distribution of the 153‐keV flux from the Medium Electrons A (MEA) experiment (Vampola et al., 1992) on the CRRES between 5.5 < *L* ^∗^<6.5 and |*λ*_*m*_|<15° for the entire mission (Figure 3). The initial differential flux was set to the median value (8.0 × 10^3^ cm^−2^·s^−1^·sr^−1^·keV^−1^) with an energy spectrum that falls rapidly with energy (i.e., the log of the flux falls linearly with energy) so that the flux at 300 keV was negligible. The electron flux on the low energy boundary was then increased rapidly by a factor of *I* = 10 so that it would exceed the 98 percentile level, followed by an exponential decay with a time constant of 72 min so that the flux drops by a factor of 10 over a period of 2.75 hr. The flux increases were repeated every 2.75 hr to agree with the most probable time between substorms (Borovsky et al., 1993). This procedure also ensured that the flux at the low energy boundary only exceeded the 98 percentile level for a few percent of the time. The flux was not allowed to exceed 10^5^ cm^−2^·s^−1^·sr^−1^·keV^−1^, which corresponds to the 99.5 percentile.

By setting the low energy boundary at 150 keV we are effectively restricting the calculation to trapped electrons that are able to drift all the way around the Earth. While the flux may vary with MLT (Allison et al., 2017) the calculation is a drift average as is done in almost all radiation belt models. To capture variations at lower energies requires a 4‐D code that includes local time variations (e.g., Shprits et al., 2015), but this is beyond the scope of our work.

## 5 Results

**Figure 4:**
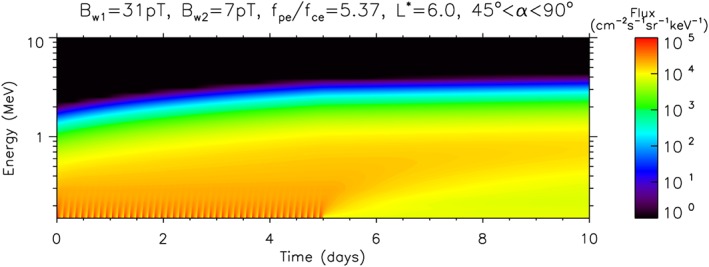
Evolution of the differential electron flux (color coded) as a function of energy and time at L ^∗^=6 due to lower‐band chorus waves. The first 5 days were run with substorm injections and a wave amplitude of B_w1_=31 pT and the following 5 days without substorms and B_w2_=7 pT.

Figure 4 shows the time evolution of the differential electron flux for geostationary orbit from a preexisting radiation belt. In these runs the initial integral electron flux *J*(*E* > 2) was set to 0 with the spectrum as described in section 4.3, and the model was run with *B*_*w*1_=31 pT. The run was stopped once *J*(*E* > 2) reached 3 × 10^2^ cm^−2^·s^−1^·sr^−1^ to represent the preexisting radiation belt. The resulting spectrum was then used as the initial condition and the model was run with *B*_*w*1_=31 pT for 5 days followed by *B*_*w*2_=7 pT for 5 days without substorm injections to represent low activity after the fast solar wind stream had passed. The differential flux was averaged between pitch angles of 45° and 90° to take into account any pitch angle anisotropy. The averaging also aids better comparison with GOES data, which have detectors with an opening angle of ±45° in elevation and ±30° in azimuth pointing westward and thus measure a wide range of pitch angles (Onsager et al., 2004).

The substorm injections are visible by the modulation at low energy but note that there is a steady buildup of the electron flux at higher energies up to several megaelectron volts. The results show that energy diffusion operates on a longer timescale than substorm injections and is a cumulative effect. After 5 days when the wave amplitude was reduced the high‐energy flux increased very slowly and remained high until the end of the simulation (day 10). The flux at lower energies decayed slowly indicating that without injections chorus waves result in a net loss of electrons less than 1 MeV into the atmosphere.

**Figure 5:**
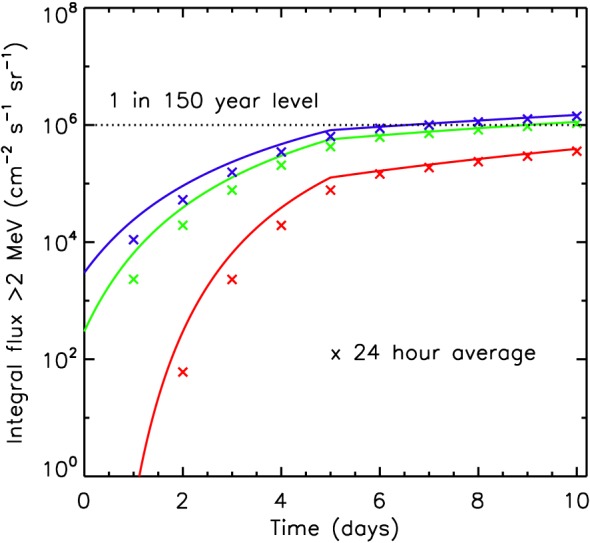
Evolution of the integral electron flux >2 MeV at L ^∗^=6 due to lower‐band chorus waves. The initial preexisting radiation belt flux was (green) 3 × 10^2^ cm^−2^·s^−1^·sr^−1^, (blue) 3 × 10^3^ cm^−2^·s^−1^·sr^−1^, and (red) no preexisting belt. The crosses show the average 24‐hr integral electron flux at the end of each 24‐hr period. The horizontal dotted line is the 1 in 150‐year flux level from an independent statistical analysis of electron data.

Figure 5 shows how the integral electron flux *J*(*E* > 2) increases with time. The initial value was set to 3 × 10^2^ cm^−2^·s^−1^·sr^−1^ (green) corresponding to a preexisting radiation belt and the runs repeated for a higher initial value (3 × 10^3^ cm^−2^·s^−1^·sr^−1^, blue) and no radiation belt (red). There is a rapid acceleration over the first few days. After 5 days when the wave amplitudes were reduced the integral flux increases very slowly and tends toward a plateau. The result is relatively insensitive to the initial value. The crosses show the 24‐hr average at the end of each 24 hr.

It is remarkable that the integral flux found from this study is comparable to that for 1 in 150‐year event found from an independent extreme value analysis of electron data from GOES West (Meredith et al., 2015). The 1 in 150‐year event is shown in Figure 5 as a horizontal dotted line at 9.9 × 10^5^ cm^−2^·s^−1^·sr^−1^. The results suggest that fast solar wind streams can lead to very high electron flux with a risk of satellite charging.

The worst‐case daily averaged electron flux greater than 2 MeV observed at geostationary orbit over the last 20 years was 4.95 × 10^5^ cm^−2^·s^−1^·sr^−1^ and occurred on 29 July 2004 (Meredith et al., 2015). This occurred just after three CME‐driven magnetic storms, moderate storms as measured by *D*_*st*_, the last of which occurred on 27 July. Therefore, the flux calculated here is only a factor of 2 higher than that which has already been observed. We therefore consider the calculation here to be a reasonable worst case.

## 6 Sensitivity Tests

**Figure 6:**
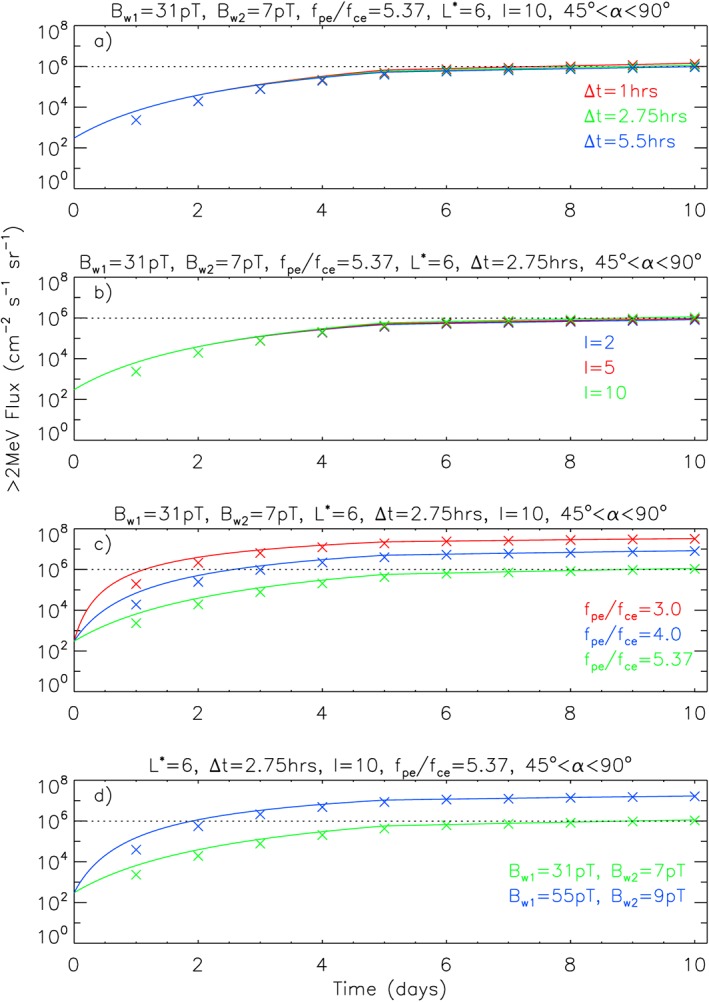
Sensitivity of the integral electron flux >2 MeV to variations in (a) the time interval between substorms, (b) the intensity of substorm injections, (c) the ratio f_pe_/f_ce_, and (d) the wave amplitude. The crosses show the average 24‐hr integral electron flux at the end of each 24‐hr period.

To test the sensitivity of the results the time between substorms was doubled to 5.5 hr and reduced to 1 hr keeping the same decay time. Figure 6a shows that the results are very insensitive. Similarly, the results are very insensitive to a reduction in the injected flux at the lower boundary from *I* = 10 to 5 and 2 (Figure 6b). We reduced the factor I rather than increased it due to the constraint that the flux should not exceed the 98 percentile level for more than a few percent of the time.

The results are much more sensitive to a reduction in the ratio f_pe_/f_ce_ (Figure 6c) or to an increase in wave amplitude (Figure 6d). In particular, the integral flux would be more than a factor of 10 higher if f_pe_/f_ce_ is reduced to 3. While lower values are possible at some locations for limited periods (Thorne et al., 2013), there is no evidence to suggest that such low values have been observed consistently for periods of 5 days or more. Furthermore such low values are not supported by our statistical analysis of the data. The flux would also be higher if we had used the root‐mean‐square amplitude for A E > 750 nT instead of the median (Figure 6d), but this would give unreasonable weight to a small number of high‐amplitude wave observations. Also, it is more likely that A E would fluctuate below 750 nT during some of the 5‐day period suggesting lower amplitudes. In the absence of further data we conclude that the flux presented here is a reasonable worst case.

## 7 Risk of Internal ESD

**Figure 7:**
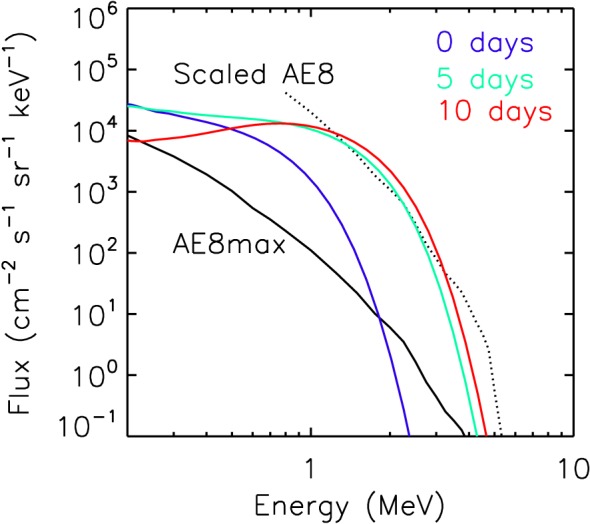
The electron spectrum (blue) at the beginning of the simulation for a preexisting radiation belt, (green) after 5 days and (red) after 10 days. Also shown is (in black) the spectrum for AE8max and (dotted) AE8 scaled according to the integrated flux greater than 2 MeV.

**Figure 8:**
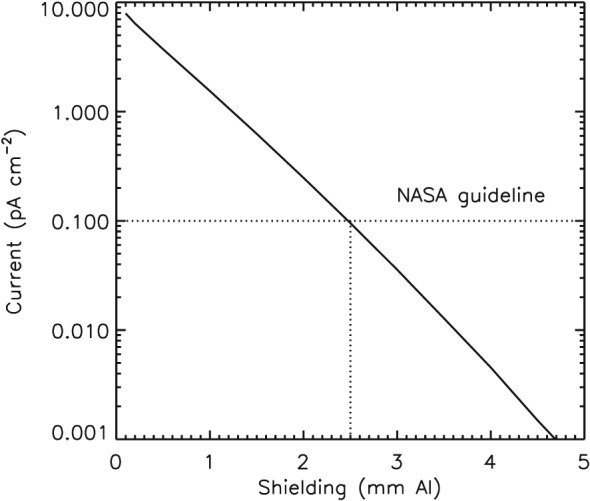
Electron current penetrating different thicknesses of aluminium shielding. The National Aeronautics and Space Administration‐recommended guideline is shown by the horizontal dotted line indicating that 2.5 mm of Al is required to reduce the current below 0.1 pA cm^−2^. NASA = National Aeronautics and Space Administration.

The NASA Technical Handbook (2011) states that if the incident electron flux is greater than 0.1 pA cm^−2^ then sensitive electronic components should be shielded so that the flux does not exceed that value. The European Cooperation for Space Standardization (ECSS) recommends shielding if the incident flux exceeds 0.02 and 0.1 pA cm^−2^ for materials in excess of 25 °C (ECSS‐E‐ST‐10‐04C, 2008). Using the DICTAT code (Sorensen et al., 2000), we calculated radiation transport through different levels of aluminium shielding. The incident electron spectrum was taken from the simulation run after 5 days (green curve in Figure 5) and is shown by the green curve in Figure 7. The initial spectrum is also shown (in blue) and that after 10 days (in red). The spectrum is somewhat higher than that from AE8 max model (shown in black). However, when the AE8 max is scaled up according to the integral flux *J*(*E* > 2) after 5 days (4.3 × 10^5^ cm^−2^·s^−1^·sr^−1^, dotted line) the shape of the simulation spectrum agrees very well with the AE8 max model. We used a planar geometry to calculate the radiation transport through different thicknesses of Al. Note that we included the entire electron spectrum that extends to lower energies. Figure 8 shows that at least 2.5 mm of Al would be required to reduce the current below the recommended NASA guideline. This is much more than that used for satellites in geostationary orbit. Typically, electronics are shielded with 1 mm of Al, with the satellite skin offering the equivalent of 0.5 mm and other components another 0.5 mm or less if the electronics are near the surface. Connectors and insulators outside the box would be more exposed. Thus, we would expect many satellites to report ESD anomalies during such an event with a strong likelihood of service outage and total satellite loss.

## 8 Discussion

One of the important omissions in our calculations is that of radial diffusion. New results from the Van Allen Probes mission show that the peak in electron phase space density generally lies inside geostationary orbit (Reeves et al., 2013), and therefore, radial diffusion should act to transport relativistic (MeV) electrons across geostationary orbit toward the outer boundary of the Earth's magnetic field (Shprits et al., 2006). However, during fast solar wind streams ULF waves are significantly enhanced (Mann et al., 2004), and thus, radial diffusion is likely to be very efficient, especially near geostationary orbit as the magnetic diffusion coefficient scales as *L* ^10^ (Brautigam & Albert, 2000). As a result the radial gradient in phase space density is likely to be flat as any deviations would be removed rapidly by radial diffusion. Indeed, flat gradients have been observed during periods of frequent particle injections (Selesnick & Blake, 1997), and this is implicit in our calculations. However, if wave acceleration is more efficient at lower *L* ^∗^ resulting in a higher flux at lower *L* ^∗^, then it is possible that outward radial diffusion could increase the flux at geostationary orbit. In this case our calculations may be an underestimate. The inclusion of radial diffusion is a challenging problem for an extreme event where there are no observations and no clear boundary conditions and will be addressed at a future stage.

Another important factor affecting these calculations is the duration of a fast solar wind stream. We have chosen a period of up to 5 days, but there remains some flexibility in this choice. However, as Figure 5 shows the integral electron flux tends toward a limiting value after 5 days, and therefore, the duration of the event is not quite such a critical parameter. If the initial flux were higher, Figure 6 suggests that the limiting value would be reached more quickly.

Electromagnetic ion cyclotron waves could contribute to electron loss at high energies, typically greater than a few megaelectron volts. However, at a few megaelectron volts the waves tend to remove electrons with small pitch angles and leave behind the flux at large pitch angles (Kersten et al., 2014; Usanova et al., 2014). Since the integral flux greater than 2 MeV is dominated by the flux near a few megaelectron‐volt electromagnetic ion cyclotron waves are more likely to create an anisotropic distribution.

In general, our ability to simulate extreme space weather events is at an early stage and is hampered by the lack of data for such events. Yet such simulation studies are important as changes in the natural environment are the starting point for any engineering assessment and wider impact assessment on critical infrastructure. In terms of basic research, more studies of the Sun, the evolution of the solar wind from the Sun to Earth, and the interaction of the solar wind with the Earth's magnetosphere are needed. Inside the magnetosphere, more studies on the different types of plasma waves and electric fields that contribute to the transport, acceleration, and loss of high‐energy electrons are needed, together with better models of electron plasma density and disruption of the geomagnetic field during disturbed times. More effort is also required to develop better simulation models, couple them together, and use them to reproduce past events as a test of our understanding. In addition, we need to transition research models to operational models so that we can develop a better forecasting capability and help better protect our critical infrastructure.

## 9 Summary and Conclusions

We have developed a model to calculate the maximum electron flux greater than 2 MeV at *L* ^∗^=6 for an extreme space weather event driven by a fast solar wind stream. The model is based on electron acceleration and loss due to wave‐particle interactions with lower‐band chorus waves, which have been shown to play a major role in the formation of the Earth's radiation belts. Electron injections due to substorms at lower energies are included by a series of rapid increases and decay of the electron flux at the low energy boundary. The approach is based on new physical understanding and is complementary to earlier statistical methods.

The main result is that for an extreme space weather event driven by a fast solar wind stream the integral electron flux greater than 2 MeV increases rapidly but tends toward a plateau. For a realistic worst‐case event lasting 5 days the integral flux can reach 10^6^ cm^−2^·s^−1^·sr^−1^, which is comparable to the level found from independent extreme value analysis of electron data at geostationary orbit (Meredith et al., 2015). The flux could remain at this level for days. To reduce the internal charging currents to below the recommended guidelines approximately 2.5 mm of Al shielding would be required, which is typically much more than that currently used. Thus, we would expect many satellites to report ESD anomalies during such an event with a strong likelihood of service outage and total satellite loss.

The results depend on a number of factors, and the resulting flux could be substantially higher if the wave amplitudes are consistently higher or the ratio *f*_*pe*_/*f*_*ce*_ is lower over a 5‐day period. However, the results are based on a statistical analysis of data from nine satellites and represent our best attempt at a reasonable worst case.

In a major geomagnetic storm, such as a Carrington‐type event, the electron flux is usually depleted at geostationary orbit and peaks much closer to the Earth (Baker et al., 2004; Bühler et al., 1998). Our simulations show that for a fast solar wind stream lasting 5 days or more the flux is significantly enhanced at geostationary orbit and can remain so for days. Thus, we conclude that satellites at geostationary orbit are more likely to be at risk from a fast solar wind stream event lasting 5 days or more than they are from Carrington‐type event. We therefore suggest that fast solar wind stream events should be recognized as a different class of extreme space weather that affects satellites in particular. As a result, more research is needed to forecast the precursors such as coronal holes on the Sun, the evolution of the solar wind from Sun to Earth, and the interaction between the solar wind and the Earth's magnetosphere in order to enable better predictions and take mitigating action.

## Appendix A The Diffusion Equation

### A.1

The 2‐D diffusion model is given by

(A.1) $$\begin{array}{cc} \frac{\mathrm{\partial f}}{\mathrm{\partial t}} & =\frac{1}{g(\alpha )}\frac{\partial }{\partial \alpha }g(\alpha )\left(D_{\alpha \alpha }\frac{\mathrm{\partial f}}{\partial \alpha }+D_{\mathrm{\alpha E}}\frac{\mathrm{\partial f}}{\mathrm{\partial E}}\right)\\ \;+\frac{1}{A}\frac{\partial }{\mathrm{\partial E}}A(E)\left(D_{\mathrm{EE}}\frac{\mathrm{\partial f}}{\mathrm{\partial E}}+D_{\mathrm{\alpha E}}\frac{\mathrm{\partial f}}{\partial \alpha }\right)-\frac{f}{\tau_{L}}, \end{array}$$

where *f* is the electron distribution function; *D*_*α**α*_, *D*_*α**E*_, and *D*_*E**E*_ are the pitch angle, mixed pitch angle‐energy, and energy diffusion coefficients defined as *D*_*α**α*_=〈*Δ* *α* *Δ* *α*〉/2*Δ* *t*, *D*_*α**E*_=〈*Δ* *α* *Δ* *E*〉/2*Δ* *t*, and *D*_*E**E*_=〈*Δ* *E* *Δ* *E*〉/2*Δ* *t*, respectively, and *τ*_*L*_ is the loss timescale due to collisions. Here *τ*_*L*_ is set to a quarter of the electron bounce time inside the loss cone and is infinite outside the loss cone. We also have

(A.2) g(α)=T(α)sin2α,

where $T(\alpha )=(1.3802-0.3198(\sin\alpha +\overset{\frac{1}{2}}{\sin}\alpha ))$ (Lenchek et al., 1961) and

(A.3) $$A(E)=(E+E_{0}){\left(E(E+2E_{0})\right)}^{\frac{1}{2}},$$

where *E*_0_ is the electron rest mass energy. The boundary conditions for the model are that the gradient of the pitch angle distribution should be 0 at *α* = 0° and 90° and that *f* = 0 at the high energy boundary at *E* = 100 MeV and vary at the low energy boundary at 150 keV as described in the text. The differential electron flux *J*(*E*,*α*) is calculated from the distribution function using

(A.4) $$J=f/p^{2}$$

and converted into units of cm^−2^·s^−1^·sr^−1^·keV^−1^.

## Appendix B Wave Model

### B.1

To calculate pitch angle and energy diffusion coefficients we used a Gaussian spectrum with parameters based on observations appropriate at geostationary orbit (Glauert & Horne, 2005; Horne, Kersten, et al., 2013; Sicard‐Piet et al., 2014). The frequency of the maximum is *f*_*m*_/*f*_*c**e*_=0.3, with a width *δ* *f*/*f*_*c**e*_=0.1 and upper and lower frequency cutoffs *f*_*u**c*_=*f*_*m*_+2*δ* *f* and *f*_*l**c*_=*f*_*m*_−2*δ* *f* outside of which wave power is set to 0. The wave normal angle distribution is also a Gaussian in *X* where X=tanψ and *ψ* is the angle between the *k* vector and the magnetic field direction. The wave normal angle distribution is peaked in the field‐aligned direction with *X*_*m*_=0, a width $X_{w}=\tan30$° (Agapitov et al., 2013; Li et al., 2011) and cutoffs *X*_*m**i**n*_=0 and $X_{\max}=\tan45\text{°}$. To obtain the bounce‐averaged diffusion rates it is also assumed that the wave power extends in latitude along the geomagnetic field up to a latitude of 30° (Horne, Kersten, et al., 2013) as the contribution of waves at higher latitudes is small. Using a dipole magnetic field model, the pitch angle and energy diffusion coefficients were calculated using the PADIE code (Glauert & Horne, 2005).
